# Bunk-Bed-Related Fractures in Children: Are We Aware of the Risks?

**DOI:** 10.3390/medicina58060749

**Published:** 2022-05-31

**Authors:** Johannes Wolfgang Duess, Ina Sorge, Martin Lacher, Peter Zimmermann

**Affiliations:** 1 Department of Pediatric Surgery, University of Leipzig, 04103 Leipzig, Germany; martin.lacher@medizin.uni-leipzig.de (M.L.); peter.zimmermann@medizin.uni-leipzig.de (P.Z.); 2 Department of Pediatric Radiology, University of Leipzig, 04103 Leipzig, Germany; ina.sorge@medizin.uni-leipzig.de

**Keywords:** bunk bed, children, mechanism, fracture, surgery

## Abstract

*Background and Objectives*: Falls from heights are a common mechanism of trauma in children. However, data on bunk-bed-related (BBR) fractures are scarce. We aimed to assess types of fractures and age groups most at risk for BBR fractures. *Material and Methods*: We analyzed medical records and imaging procedures of patients aged <18 years who sustained a bunk bed injury and were treated at our department between January 2014 and December 2021. Demographic data, including age groups, mechanisms, types and anatomical regions of fractures, were assessed. *Results*: A total of 162 patients (median age 5 years, range 0–15; 59.9% male) was included. Fractures were recorded in 80 (49.4%) and contusions and abrasions in 49 (30.2%) cases. BBR fractures were recorded in 44.8% of children below the age of 3, in 50.8% aged 3–5, in 58.5% aged 6–9 and in 28.6% ≥ 10 years. Forearm fractures were most common (n = 34, 42.5%), followed by fractures of the clavicle (n = 13, 16.3%), humerus (n = 10, 12.5%), foot (n = 8, 10.0%), hand (n = 5, 6.3%), lower leg (n = 5, 6.3%) and skull (n = 5, 6.3%). Surgery was required in 12 (15.0%) cases, including closed reduction (n = 7) and closed reduction with internal fixation (n = 5). Overall, 21 (26.3%) patients were hospitalized with a mean length of stay of 2 ± 1.6 days. *Conclusions*: Caregivers should be aware that bunk beds cause a significant amount of severe trauma in children and adolescents, especially in those younger than 10 years of age. Caregivers would benefit from receiving information about these risks and evidence-based strategies to prevent BBR fractures.

## 1. Introduction

Falls from heights are a common mechanism of trauma in the pediatric population, leading to visits to emergency room departments in most cases [1,2,3]. For example, an estimated 23,000 children up to 9 years of age are treated annually in the US for bunk bed injuries [2]. Bunk beds are widely used in homes across industrialized countries and are often introduced in response to space-saving needs in crowded urban dwellings [4,5]. Although some mandatory product safety standards such as specific guardrails have been established, severe injuries are still frequently reported [2,5]. The US Consumer Product Safety Commission has recommended cautionary labelling to prohibit children less than 6 years of age from using the upper bunk. However, previous reports have shown that children in this age group still sustain the majority of bunk-bed-related (BBR) injuries [1,2]. Fractures associated with BBR falls account for up to 40% of all injuries, affecting the upper extremities in two-thirds of cases. These children are almost six times more likely to require hospitalization [1,2]. However, data on BBR fractures are generally scarce.

The aim of this retrospective study is to assess types of fractures and age groups most at risk for BBR fractures.

## 2. Materials and Methods

The study was performed at the Department of Pediatric Surgery at the University hospital Leipzig, which serves the state of Saxony (Germany) as a tertiary pediatric trauma center. In a period from January 2014 to December 2021, all medical records and imaging procedures of patients aged <18 years who sustained a bunk bed injury were retrospectively reviewed. Patients without imaging procedures were excluded.

Detailed information was collected on demographic data, mechanism of injury, types and anatomical regions of fractures, methods of treatment, as well as length of hospital stay. To analyze which age groups are at most risk, patients were divided into four different age groups: <3 years, 3–5 years, 6–9 years and ≥10 years.

Data are shown as median (range) or number of cases (%).

## 3. Results

A total of 162 patients with a BBR injury, in whom radiologic imaging was performed, was identified. The median age was 5 years (0–15). Male patients accounted for 97 (59.9%) cases. Twenty-nine children (17.9%) were below the age of 3 years, fifty-nine patients (36.4%) were aged 3 to 5, fifty-three (32.7%) between 6 and 9 years and twenty-one children (13.0%) aged ≥ 10 years (Table 1).

Falls from bunk beds were the most common mechanism of injury (n = 134, 82.7%), while the remaining injuries were caused by falls from the ladder (n = 14, 8.6%) or jumps (n = 14, 8.6%) from the bunk bed.

Fractures were found in 80 children (49.4%), including 48 (60.0%) males (Table 2). Contusions/abrasions were recorded in 49 cases (30.2%), 26 children (16.0%) presented with head injuries and lacerations were found in 5 cases (3.1%). In children with fractures, the upper extremity was affected in 62 (77.5%) and the lower extremities in 14 patients (17.5%). Forearm fractures were the most common injury (34 patients, 42.5%), followed by fractures of the clavicle in 13 (16.3%) and humeral fractures in 10 children (12.5%). Fractures of the foot were recorded in eight patients (10.0%) and of the hand as well as the lower leg in five (6.3%) cases each. One boy presented with a femur fracture (1.3%). Five children (6.3%) aged 10 months to 8 years sustained skull fractures. One girl had a combined fracture of her humerus and forearm (Table 2). A total of 21 children (26.3%) with BBR fractures required hospitalization, with a mean length of stay of 2 ± 1.6 days. Patients with head injuries or skull fractures did not show any neurological deficits during hospitalization and follow-up appointments shortly after discharge. Twelve children (15.0%) required surgery, ten of the upper and two of the lower limbs, including closed reduction in seven and closed reduction with internal fixation in five patients.

An analysis of the different age groups showed that BBR fractures occurred in 13 children (44.8%) below the age of 3 and in 30 (50.8%) between 3 and 5 years. In children aged 6–9 years, which comprises the majority of primary school children, BBR fractures were recorded in 31 (58.5%) cases. Six children (28.6%) were ≥10 years of age (Table 3). Hence, 43 patients with fractures were younger than 6 years of age, accounting for 53.8% of all fractures recorded in our cohort. Including the age group of 6–9 years, a total of 74 was younger than 10 years of age, representing 92.5% of all BBR fractures.

## 4. Discussion

Bunk beds are often considered as inevitable for space-saving requirements, mostly in urban dwellings [2]. Due to severe trauma in children associated with bunk beds, standards were introduced decades ago [1,5]. However, previous reports have shown severe BBR injuries. We designed this retrospective study to specifically investigate BBR fractures in a tertiary trauma center.

### 4.1. Demographics

We identified 162 patients who underwent an imaging procedure following a BBR injury. Male patients accounted for 59.9% of all cases, which corresponded with the literature [2,3,4,5,6,7,8].

The prevalence of fractures following a bunk bed injury has been reported to range from 10% to 40%; however, papers specifically focusing on BBR fractures in detail are still scarce [1,3,7]. We found that 49% of patients in our cohort sustained a fracture. In contrast to most recent publications analyzing all different types of BBR injuries, we only included children on whom an imaging procedure was performed and, therefore, our numbers of fractures were higher and could not be compared to previous publications. We found that 78% of fractures affected the upper extremities, which could be compared to findings described by D’Souza et al. in 2008. The authors analyzed 572,580 children and adolescents aged ≤21 years who presented to the emergency department for BBR issues, and 19.9% of those patients sustained a fracture with 68% affecting the upper extremities [1]. In addition, McFaull et al. described the Canadian experience with emergency department presentations for BBR injuries from 1990 to 2009 and reported a fracture rate of 40%. Out of 934 upper bunk-related incidents, 411 affected the upper extremities and 340 lead to a fracture, resulting in over 80% of all recorded cases in that subgroup [3]. In our cohort, forearm fractures were the most common (43%), followed by fractures of the clavicle (16%), humerus (13%), foot (10%), hand (6%), lower leg (6%) and skull (6%), as well as the femur in one child (1%) (Table 2). As already discussed in previous papers on bunk bed injuries, the increased risk for fractures of the upper extremities might be due to physiological attempts to absorb a fall using out-stretched arms, whereas jumps from the bunk bed were more likely to result in lower-extremity injuries, more likely causing strains and sprains [7].

### 4.2. Hospitalization and Surgical Intervention

Twenty-one children (26%) required hospitalization, which was higher than described in other studies. However, these statistics might be difficult to compare, as each institution has different admission policies [3]. Mack et al. investigated BBR injuries in children aged 0–9 years in the US from 2001 to 2004, a cohort which was updated by D’Souza et al. in the following year, and found a fracture rate of 28%, of which 8.7% needed hospitalization.

Surgery was performed on twelve children (15%) in our cohort, including closed reduction in seven and closed reduction with internal fixation in five cases. The upper limbs were affected in ten of those twelve cases. In general, fractures to the upper extremities often require immediate surgical intervention due to the extensive nerve and vascular supply in that region, followed by hospitalization [1].

### 4.3. Skull Fractures

Besides fractures to upper or lower limbs, we also detected five skull fractures (6%), which was in line with Mayr et al., who found a similar percentage of skull fractures. Out of 218 children, 7 (3.2%) sustained a skull fracture without neurological deficits or morbidity after the trauma [5]. In general, due to a higher center of gravity, small children tend to fall head first, leading to injuries such as concussions and skull fractures [1,2,8,9]. In addition, Sawyer assessed fracture patterns associated with falls from heights, and described that infants fall head first and children fall feet and/or hands first, whereas adolescents and adults fall feet first [10]. However, we could not reveal this pattern in terms of skull fractures in our cohort, as two children were below the age of three and the other three older than six years of age.

### 4.4. Age Groups Affected by Fractures

The majority of reports indicate that the most vulnerable group of patients is below the age of six, when considering different BBR injuries [1,2,3,4,7,11]. In the large study conducted by D’Souza et al. over a 16-year period in the US, almost half of the children injured were also younger than 6 years [1]. A similar finding was reported by McFaull when investigating the Canadian cohort, stating a peak of incidents in the 3–5-year age group [3]. In contrast, more than 90% of all children in this study who sustained a fracture were below the age of 10 years, with the highest rate in patients aged 6–9 years (58.5%), which includes the majority of primary school children (Table 3). However, the fact that we only included patients who required an imaging procedure to investigate suspected BBR fractures and did not focus on injuries such as superficial lacerations, contusions, abrasions, concussions as well as strains/sprains, might be an explanation for this finding. In addition, Mack et al. also reported in their cohort that the highest rate of fractures (34.4%) was found in this specific age group of 6–9 years [2].

### 4.5. Safety Standards

Fortunately, we did not observe any mortality following a bunk bed injury in our cohort. However, some authors did report deaths, mainly due to entrapment and poor standards of beds [5]. In recent decades, multiple product safety standards have been established by consumer product safety commissions in different countries, such as the requirement of at least two upper bunk guardrails which run continuously on the side opposite to a ladder. It is also suggested to have a carpeted floor and to use mattresses recommended by the manufacturer to ensure a sufficient height to prevent falls from the upper bunk. Next, ladders should always be fixed to the bed, but most importantly, children under the age of six should be prohibited from sleeping in the upper bunk [12,13]. When considering this age range only, more than half of all BBR fractures recorded in our cohort could have been prevented. Therefore, considering that most BBR falls occur at home, all safety standards should be clear and understood by caregivers to avoid severe injuries and possible associated morbidities.

### 4.6. Limitations

We are aware of the limitations of this single-center retrospective study. Due to the fact that only patients who received radiologic imaging were included, a comprehensive amount of all BBR injuries, including skin lacerations, head injuries, contusions, abrasions, tooth injuries or hematomas, was not represented in this study. However, we believe that the high number of fractures found in this subgroup of patients does reflect the extent of trauma caused by bunk beds.

## 5. Conclusions

In conclusion, the majority of children younger than 10 years of age included in this retrospective study sustained a fracture following a bunk bed injury. Caregivers would benefit from receiving information about these risks and evidence-based strategies to prevent BBR fractures.

## Figures and Tables

**Table 1:** BBR injuries in different age groups (0–<18).

Age Group (Years)	Bunk Bed Injuries (n = 162)	
<3	29	17.9%
3–5	59	36.4%
6–9	53	32.7%
≥10	21	13.0%

**Table 2:** Type and rate of BBR fractures (one child sustained a combined fracture of the humerus and forearm).

Site of Fracture	n = 80 *	%	<3 Years	3–5 Years	6–9 Years	≥10 Years
Forearm	34	42.5	6	12	14	2
Clavicle	13	16.3	1	6	6	0
Humerus	10	12.5	0	3	6	1
Foot	8	10.0	2	5	1	0
Hand	5	6.3	0	1	2	2
Lower leg	5	6.3	2	2	0	1
Skull	5	6.3	2	0	3	0
Femur	1	1.3	1	0	0	0

* Out of a total of 162 BBR injuries.

**Table 3:** BBR fractures in different age groups. The percentage indicates the number of fractures out of all bunk bed injuries in each age group.

Age Group (Years)	Fractures (n = 80) *	
<3	13	44.8%
3–5	30	50.8%
6–9	31	58.5%
≥10	6	28.6%

* Out of a total of 162 BBR injuries.

## Data Availability

Not applicable.
